# Transfer learning for accurate fetal organ classification from ultrasound images: a potential tool for maternal healthcare providers

**DOI:** 10.1038/s41598-023-44689-0

**Published:** 2023-10-20

**Authors:** Haifa Ghabri, Mohammed S. Alqahtani, Soufiene Ben Othman, Amal Al-Rasheed, Mohamed Abbas, Hassan Ali Almubarak, Hedi Sakli, Mohamed Naceur Abdelkarim

**Affiliations:** 1 MACS Laboratory, National Engineering School of Gabes, University of Gabes, 6029 Gabès, Tunisia; 2 Radiological Sciences Department, College of Applied Medical Sciences, King Khalid University, 61421 Abha, Saudi Arabia; 3 BioImaging Unit, Space Research Centre, Michael Atiyah Building, University of Leicester, Leicester, LE17RH UK; 4 PRINCE Laboratory Research, ISITcom, Hammam Sousse, University of Sousse, Sousse, Tunisia; 5 Department of Information Systems, College of Computer and Information Sciences, Princess Nourah bint Abdulrahman University, P.O. Box 84428, 11671 Riyadh, Saudi Arabia; 6 Electrical Engineering Department, College of Engineering, King Khalid University, 61421 Abha, Saudi Arabia; 7 Division of Radiology, Department of Medicine, College of Medicine and Surgery, King Khalid University (KKU), Abha, Aseer Saudi Arabia; 8 EITA Consulting, 5 Rue Du Chant des Oiseaux, 78360 Montesson, Montesson France

**Keywords:** Computational biology and bioinformatics, Diseases, Health care

## Abstract

Ultrasound imaging is commonly used to aid in fetal development. It has the advantage of being real-time, low-cost, non-invasive, and easy to use. However, fetal organ detection is a challenging task for obstetricians, it depends on several factors, such as the position of the fetus, the habitus of the mother, and the imaging technique. In addition, image interpretation must be performed by a trained healthcare professional who can take into account all relevant clinical factors. Artificial intelligence is playing an increasingly important role in medical imaging and can help solve many of the challenges associated with fetal organ classification. In this paper, we propose a deep-learning model for automating fetal organ classification from ultrasound images. We trained and tested the model on a dataset of fetal ultrasound images, including two datasets from different regions, and recorded them with different machines to ensure the effective detection of fetal organs. We performed a training process on a labeled dataset with annotations for fetal organs such as the brain, abdomen, femur, and thorax, as well as the maternal cervical part. The model was trained to detect these organs from fetal ultrasound images using a deep convolutional neural network architecture. Following the training process, the model, DenseNet169, was assessed on a separate test dataset. The results were promising, with an accuracy of 99.84%, which is an impressive result. The F1 score was 99.84% and the AUC was 98.95%. Our study showed that the proposed model outperformed traditional methods that relied on the manual interpretation of ultrasound images by experienced clinicians. In addition, it also outperformed other deep learning-based methods that used different network architectures and training strategies. This study may contribute to the development of more accessible and effective maternal health services around the world and improve the health status of mothers and their newborns worldwide.

## Introduction

Artificial intelligence (AI) has emerged as a powerful tool for enhancing various domains, from healthcare and finance to manufacturing and transportation^1^. AI refers to the ability of machines to perform tasks that typically require human intelligence, such as recognizing patterns, problem-solving, and understanding language. With the help of AI, computers can analyze large amounts of data and make predictions and decisions based on patterns and insights that would be difficult for humans to discern. In the domain of healthcare, AI has the potential to revolutionize the way to diagnose, treat, and care for patients. By analyzing patient data, such as electronic health records and medical images, AI can help healthcare professionals to make more accurate diagnoses, develop personalized treatment plans, and improve patient outcomes. For example, AI can identify patterns in medical images, such as X-rays, CT scans, and ultrasounds, that might indicate the presence of a particular disease, such as cancer. It can also analyze electronic health records to identify risk factors for conditions such as diabetes or heart disease^2–5^ and recommend personalized treatment plans based on a patient's specific needs.

AI has the potential to bring about significant changes in the field of medical condition diagnosis by using medical images. Medical imaging is a set of techniques used to obtain images of the body’s interior structures for diagnostic and therapeutic purposes. It includes various imaging methods such as X-ray, CT, MRI, ultrasound, and PET. Each modality uses different principles and techniques to create images of body organs and tissues, making them useful for different diagnostic applications. X-ray and CT imaging use ionizing radiation to create images of bones and tissues, while MRI uses magnetic fields and radio waves to create images of organs and tissues. Ultrasound uses high-frequency sound waves to create images of internal organs and tissues and is often used for pregnancy monitoring and diagnosis of conditions such as gallbladder diseases and heart diseases. PET imaging is a nuclear medicine technique that uses radioactive tracers to create images of the body's metabolic activity. The continuous development and progress of medical imaging technology have greatly improved the accuracy of diagnosis and the effect of treatment. In this paper, we focus on ultrasound medical image modeling to detect and classify materno-fetal plans. Ultrasound is a non-invasive medical procedure that uses sound waves to create images of the inside of the body. It is commonly used to visualize the organs, muscles, tendons, and other structures inside the body, and is especially well-known for its use in monitoring the growth of a fetus during pregnancy^6,7^. However, in developing countries, regular diagnosis of pregnant women is rare due to a lack of gynecologists, which can increase the high mortality rate^8^ due to not paying attention to the fetus's development. Additionally, the low imaging quality, low contrast, and high variability of ultrasound images can make it difficult for healthcare professionals to interpret all the features of the images, making the presence of an intelligent system that assists in making clinical decisions essential. The use of AI algorithms can assist healthcare professionals in analyzing medical images to identify abnormalities or diseases. AI can recognize patterns and make predictions based on the data from medical images, which can help healthcare professionals to develop more accurate diagnoses and treatment plans. However, it is important to note that AI is not a replacement for human healthcare professionals, but rather a means of enhancing their capabilities. AI can also be used to automatically extract information from medical images, such as measurements of organ sizes or the presence of certain features, reducing the time and effort required to manually analyze images and improving the accuracy of diagnoses.

Several scientific studies have been conducted to improve the quality of prenatal diagnoses by focusing on three major issues:

1. detection of anomalies, fetal measurements, scanning planes, and heartbeat.
2. segmentation of fetal anatomy in ultrasound images and videos.
3. classification of fetal standard planes, congenital anomalies, biometric measures, and fetal facial expressions.

In the field of gynecology, the use of ultrasound technology is a critical tool for monitoring fetal development and diagnosing potential difficulties during pregnancy. The classification of maternal–fetal standard plans is an important use of ultrasound in medicine. In this paper, we propose an AI algorithm that is trained to recognize materno-fetal plans in medical ultrasound images. The proposed algorithm is based on Convolutional Neural Network (CNN) architecture, which is a type of neural network commonly used in image processing and computer vision applications^9,10^. This study introduces a novel approach to detecting materno-fetal plans by applying CNN architecture to ultrasound images from various regions, including Africa and Europe, and different scanning machines. The significance of this approach lies in its potential to develop a more reliable and precise model for identifying materno-fetal plans, which is vital for the early detection and treatment of pregnancy-related complications. The model's ability to learn from diverse images can enhance its adaptability and applicability in real-world scenarios by identifying consistent patterns and features across different settings. This study has the potential to enhance maternal healthcare by improving the accuracy and accessibility of maternal health services.

The structure of this paper is organized as follows. Section “Related work” presents a review of relevant literature on the topic. Section “Materials and methods” provides detailed information on the proposed architecture, including the model training process and parameters used. The findings of the proposed model are presented in section “Results and discussion”. Finally, in section “Conclusion”, the significance of the proposed algorithm's potential applications is discussed, along with potential future research directions.

## Related work

One of the branches of AI is machine learning, which involves computer processes that enable machines to learn from their experiences^11^. Within the field of machine learning, there is a topic called deep learning, which focuses on utilizing artificial neural networks to learn through a hierarchical structure of concepts. These neural networks are particularly useful when dealing with large datasets. The utilization of artificial intelligence (AI) tools is becoming increasingly prevalent in clinical research due to their success in prediction and categorization. As a result, they are now widely employed in biomedical investigations and the development of reliable diagnostic systems^12–15^.

Several research studies have explored the use of ultrasound in the classification of maternal–fetal standard planes. For example, in a study by Yang et al.^16^, researchers examined the use of a novel three-dimensional (3D) ultrasound technique for the classification of standard planes. The study demonstrated that the 3D technique was highly accurate in identifying fetal standard planes. Zhang et al.^17^proposed an automatic image quality assessment scheme based on multitask learning to assist in fetal sonographic image quality control. The scheme uses three convolutional neural networks to identify essential anatomical structures and judge whether a fetal sonographic image meets the standard. The results showed an accuracy of 94.3% and a precision of 94.6%. The study of Yu et al.^18^ proposed a deep convolutional neural network (DCNN) method for automatically recognizing fetal facial standard plane in prenatal ultrasound imaging. Traditional approaches have had difficulty with the high intra-class variation and visual similarity between fetal facial standard plans and non-fetal facial standard plans. The proposed DCNN architecture with transfer learning and tailored data augmentation techniques significantly improves recognition performance compared to traditional approaches. The study used a training dataset of 4 849 ultrasound images annotated by experienced obstetricians. The model exhibited a mean AUC of 0.99 and achieved high values for Accuracy, Precision, Recall, and F1, with scores of 0.96, 0.96, 0.97, and 0.97, respectively. Similarly, Qu et al.^19^ propose a differential convolutional neural network (differential-CNN) to automatically identify six fetal brain standard planes from non-standard planes. The differential-CNN framework uses differential operators to derive additional differential feature maps from the feature maps in the original CNN, which results in good identification performance and cost no extra computational burden. The method was tested on a dataset of 30,000 2D ultrasound images from 155 fetal subjects ranging from 16 to 34 weeks and achieved accuracy, precision, recall, and F1 of 0.93, 0.93, 0.92, and 0.93, respectively. Kong et al.^20^ used a multi-scale dense network to detect the fetal heart, fetal abdomen, fetal brain, and fetal facial on a testing set of 5678 ultrasound images, with Precision, Recall, and F1 values of 0.98, 0.98, and 0.98, respectively. Liang et al.^21^ used an automatic method for recognizing detect fetal heart, fetal abdomen, fetal brain, and coronal fetal facial in prenatal diagnosis using a network called SPRNet. The network is based on DenseNet and trained with fetal ultrasound images and placenta ultrasound images using data-based partial transfer learning. The results show that SPRNet achieves 0.99, 0.96, 0.99, and 0.95 for accuracy, recall, specification, and F1. Montero et al.^22^ utilized a generative adversarial network (GAN) to enhance fetal brain classification using ResNet, which was validated using 2249 pictures and yielded an AUC of 0.86 and Accuracy and F1 of 0.81 and 0.80, respectively. Meng et al.^23^ performed cross-device categorization of six anatomical standard planes, including fetal heart, fetal abdomen, and lips, by applying enhanced feature alignment to extract discriminative and domain-invariant features across domains. The results showed an average F1, Recall, and Precision of 0.77, 0.77, and 0.78, respectively. Most of the datasets for model classification based on ultrasound images are private, since, the annotation of ultrasound data is a tedious task because ultrasound images suffer from speckle noise and low contrast, in addition, ultrasound images might vary significantly due to variations in imaging machines and settings, Therefore, the creation of annotated ultrasound datasets requires significant expertise and resources, making them difficult to obtain. The limitation of the previous studies is the used dataset which only includes fetal subjects from a specific age range (16–34 weeks) and from (20–36 weeks) and may not be representative of all stages of fetal development. Some studies focus on the identification of one fetal standard plan. This may not apply to other types of ultrasound scans. Other studies have explored the use of ultrasound in the classification of maternal–fetal standard planes in specific clinical scenarios, such as the detection of fetal gender^24^ and the estimation of fetal gestational age^25,26^. In summary, various research has demonstrated that ultrasound may be used to classify maternal fetal standard plans, with good results for both 2D and 3D approaches, as well as the application of AI algorithms. Typically used for automated diagnosis, screening, or staging. In this article, we propose a model that helps doctors with the automated detection of fetal plans during screening pregnant.

Table 1 presents a comparison of the related works presented in the section focusing on the classification of ultrasound images.

**Table 1:** Related works in ultrasound image classification.

References	Plane	Model	Preprocessing	Accuracy	F1-score
Zhang et al.^17^, 2021	1 plan	CNN	No	0.94	0.94
Yu et al.^18^, 2018	1 plan	DCNN	No	0.96	0.97
Qu et al.^19^, 2020	6 standard plans	differential-CNN	No	0.93	0.93
Kong et al.^20^, 2018	4 plans	multi-scale dense network	Yes	–	0.98
Liang et al.^21^, 2019	4 plans	SPRNet	Yes	0.99	0.95
Montero et al.^22^, 2021	1 plan	ResNet	Yes	0.81	0.81
Meng et al.^23^, 2020	6 standard plans	DNN	No	–	0.77

## Materials and methods

**Figure 1:**
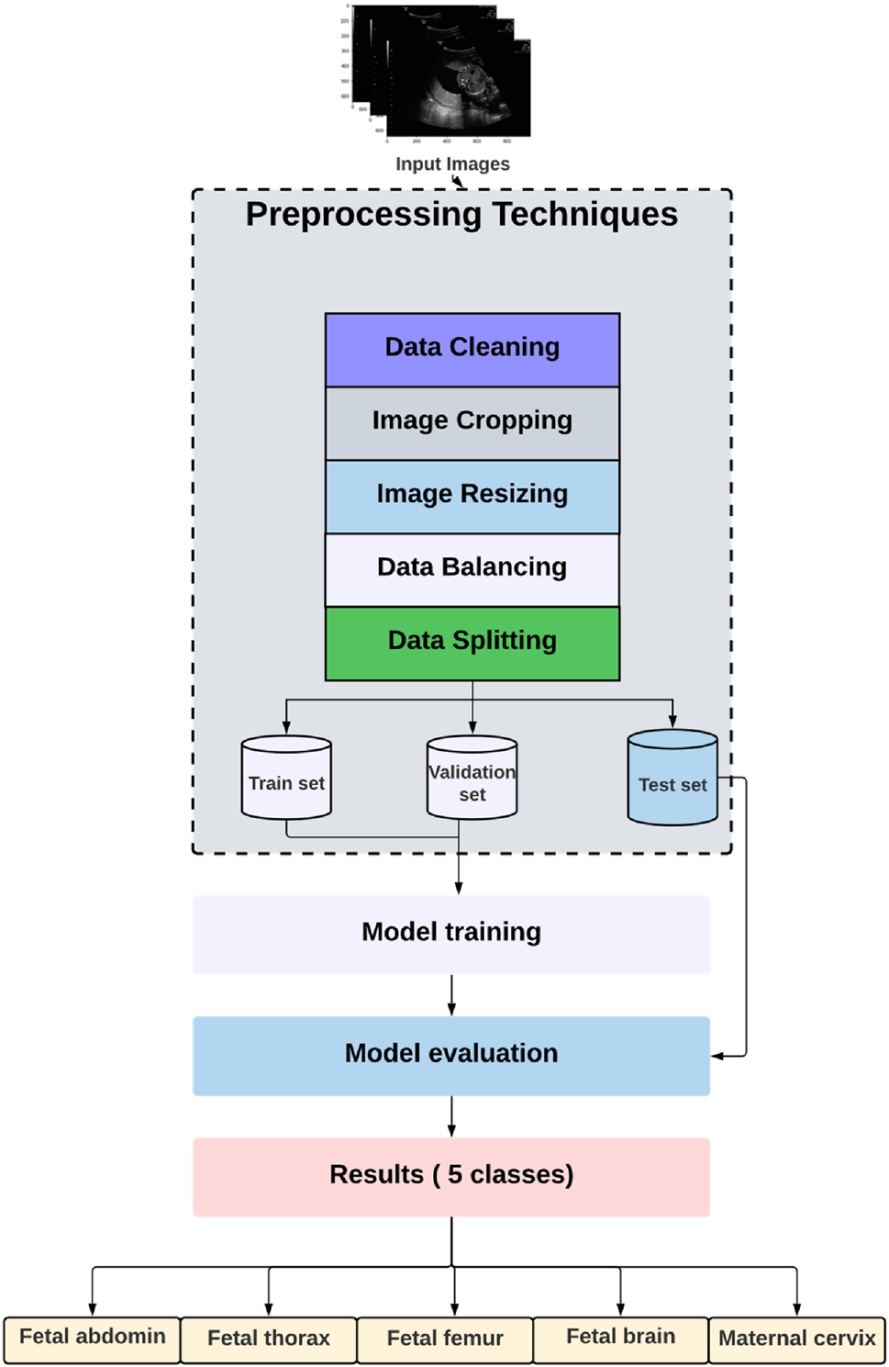
The strategy of the work is to classify fetal organs from ultrasound Images.

In this study, we aim to classify fetal plans using ultrasound image data. The proposed research method is as follows: first, we get data from a free source, which comprises various parts of materno-fetal ultrasound images, then we employ different approaches for data preprocessing since the data contain varied noise and unused information. The next step is model training, which involves implementing a different CNN architecture as a baseline for training the data to identify as a result the materno-fetal standard plans. Figure 1 summarizes the steps used in this study.

### Data

**Figure 2:**
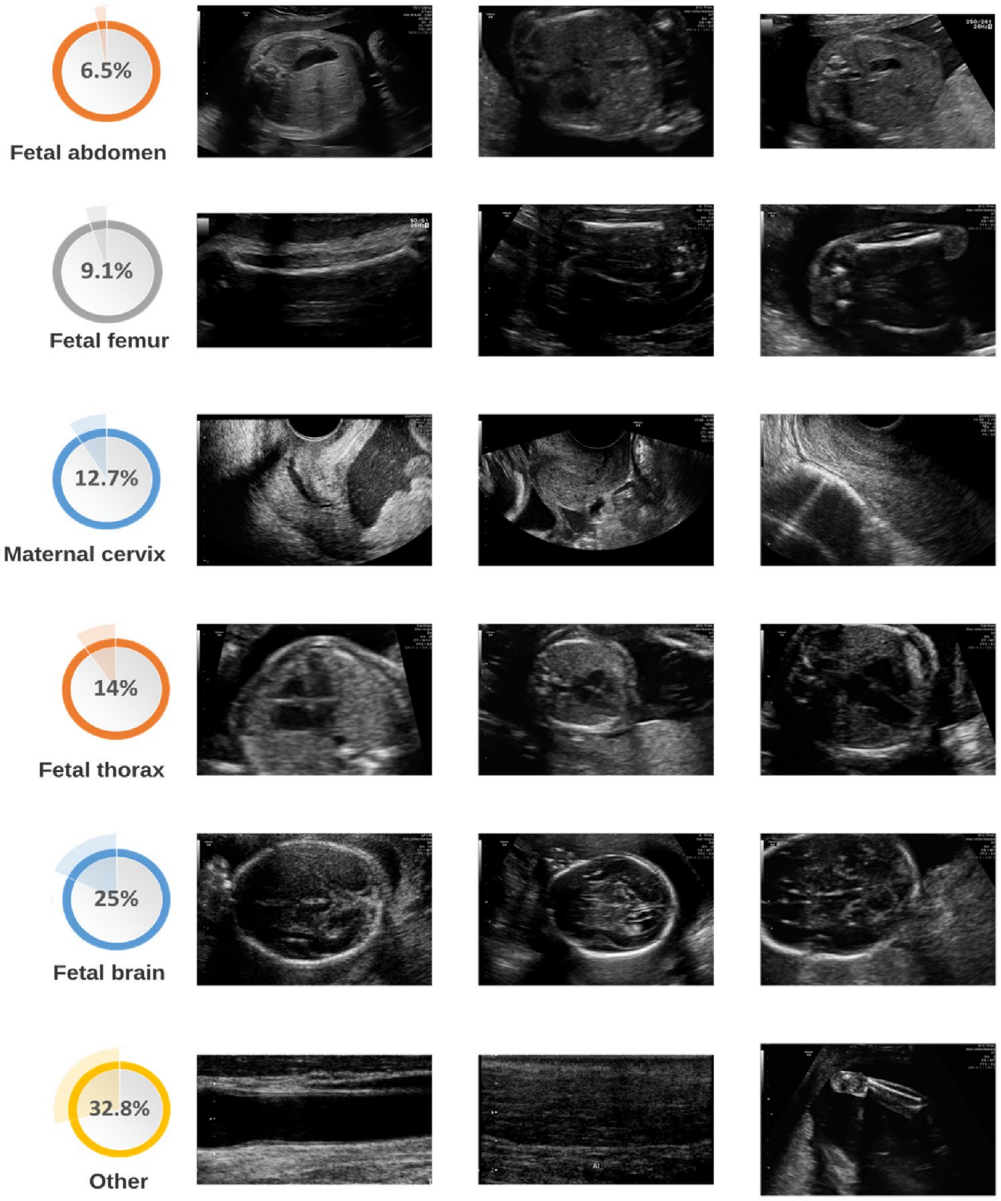
Sample from classes in the dataset for materno-fetal organ.

Obtaining a medical ultrasound dataset is a more demanding task than obtaining other types of datasets. This is due to annotating medical scans requiring specialized medical knowledge, which results in it being both scarce and costly to perform. Secondly, medical data is usually confidential, and therefore, cannot be openly shared with the public, and also training a deep learning model based on neural networks is particularly useful when dealing with large datasets. To work around these constraints, we opted to combine two publicly available datasets^27^^28^ of routinely obtained materno-fetal screening images from Spain and different countries in Africa. The first dataset was collected in 2020 from different hospitals in Barcelona Spain, the data includes 12,400 2D images from 896 pregnant women, divided into six categories: maternal cervix, thorax, femur, abdomen, brain, and other. A specialist fetal doctor manually annotated the images. The second dataset collected in 2023 contains 400 images from underdeveloped countries in Africa, namely Egypt, Algeria, Uganda, Malawi, and Ghana. the proposed approach aims to leverage the benefits of data diversity and increase the size of the training dataset. This, in turn, can lead to more robust and accurate models, which can identify various fetal plans and can be effectively deployed in different countries. The proposed combined dataset is expected to contribute to the advancement of prenatal healthcare and the detection of fetal. In Fig. 2, we illustrate a representation of the distribution of classes within the proposed database. Furthermore, we present select samples from each class, offering a detailed view of the dataset's composition across different developmental phases.

### Preprocessing

Image preprocessing is a crucial step in learning image-based AI models. Without preprocessing, AI models may perform less well or even be unable to learn from poor-quality raw data. Therefore, image pre-processing is a necessary step to obtain efficient AI models in applications such as object recognition, image segmentation, face detection, and image classification. Image preprocessing consists of a series of techniques to improve the quality of the input data by removing noise, normalizing brightness and contrast levels, correcting distortions, cropping, and resizing images.

#### Data cleaning

**Figure 3:**
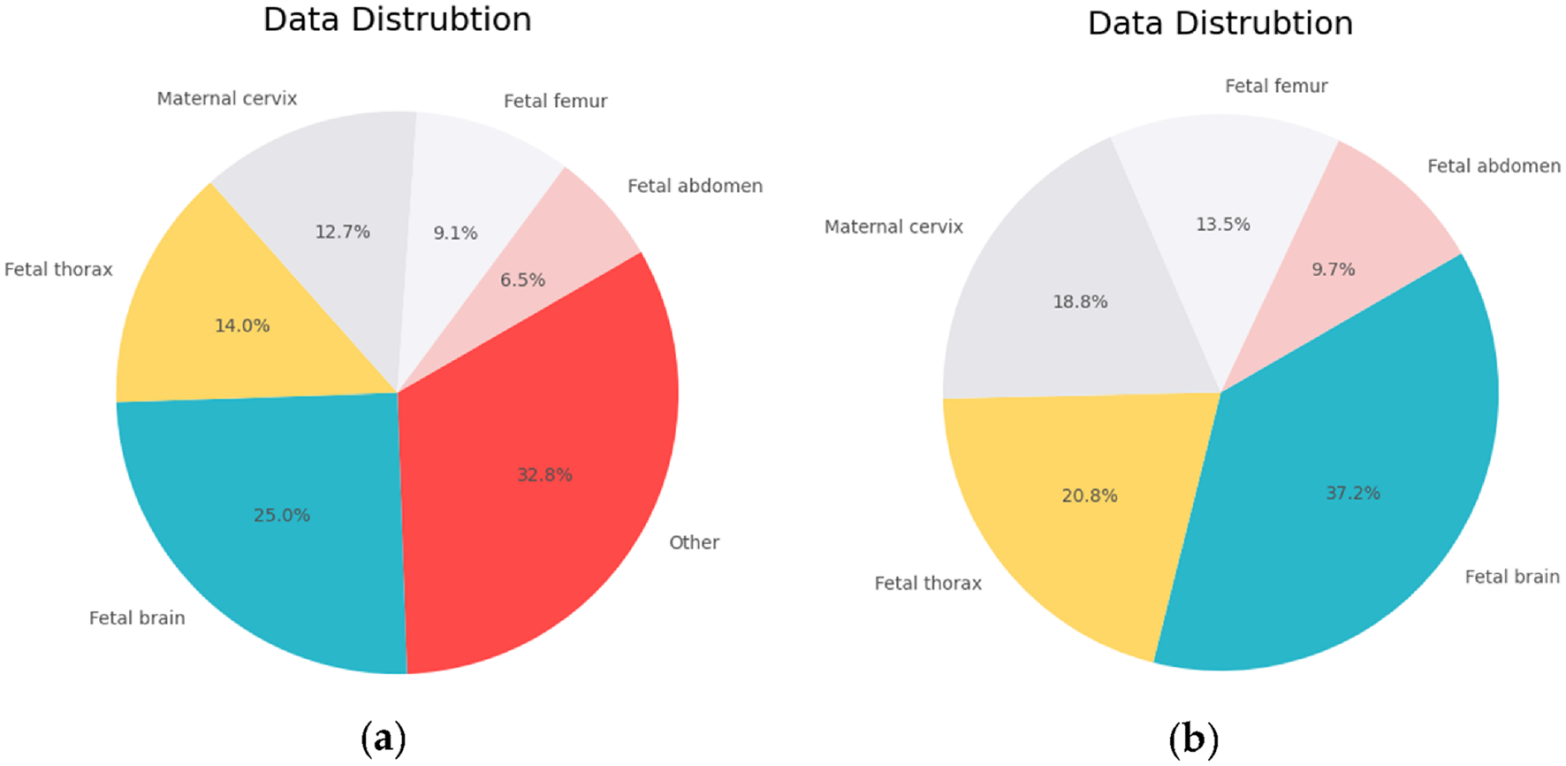
The distribution of the data with the proportion of each class in the dataset used. The visualization encompasses both (**a**) the distribution of all classes and (**b**) the distribution of classes after dropping other annotations.

Data cleaning is an important step that includes identifying and fixing errors, inconsistencies, and unnecessary data in a dataset. The purpose of the cleaning is to guarantee that the data is accurate, full, and consistent, allowing the proposed model to derive valid conclusions from it. Considering that other annotation class occupies a large proportion of the dataset compared with the rest classes, hence, we remove other annotation as a first step in the data proposed to allow the model to learn from stable input data, which improves its ability to generalize and provide accurate predictions on new data. Figure 3 presents the data distribution in the dataset used.

Algorithm 1 explain the processes of eliminate labels with value 'Other' as an annotation.


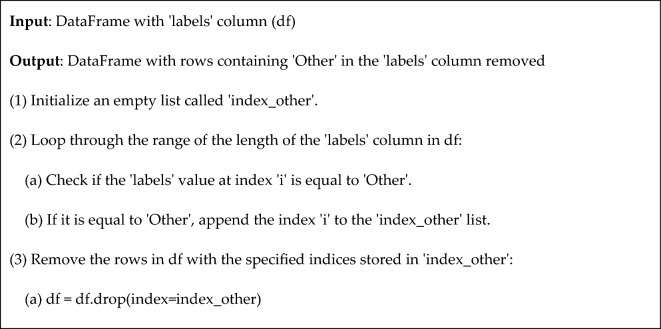


#### Data cropping

**Figure 4:**
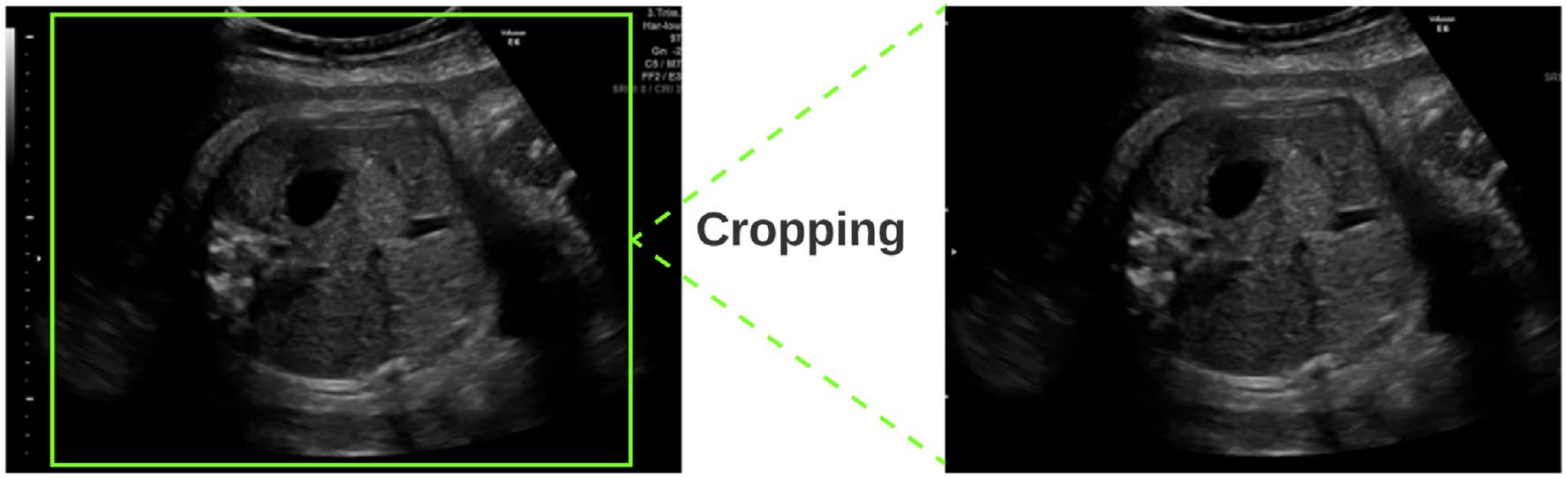
Cropping technique used in this work to eliminate unnecessary information.

Cropping images is a frequent technique for removing confusing elements and focusing on the region of interest. Images in the proposed dataset contain too much irrelevant or unnecessary information that detracts from the region of interest such as system settings, patient information, image orientation, and so on. Cropping can enhance model accuracy and make it easier for training to read the image. It is a simple and efficient approach to improve the quality of images, providing the proposed models with more useful and clearer information. By removing this unnecessary information, our cropping method improves the precision of our models and accelerates the training process. This simple and effective method significantly enhances image quality, providing more relevant and clear data to the proposed models. Furthermore, cropping is used to concentrate the model’s attention to the most important parts of an image, resulting in more precise outcomes. Figure 4 illustrates how our cropping method successfully removes unrelated and confusing information, allowing the model to perform more effectively.

Algorithm 2 explains our cropping process, which consists of the following steps: we get the image edges’ height (h) and width (w). The cropping boundaries are then defined by ensuring that the values of x and y are within acceptable ranges. The cropping is then applied using the computed values for x and y, as well as the image dimensions (h and w). Finally, as output, we return the cropped image (cropped_edges).


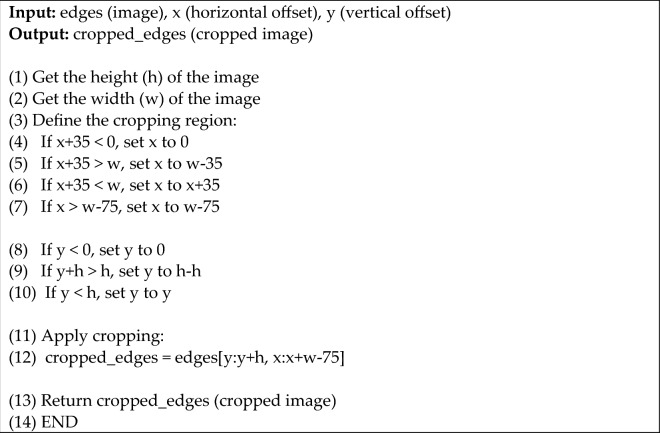


#### Data balancing

To improve our database entry, we applied data augmentation techniques to increase the representation of minority classes in the dataset.

**Figure 5:**
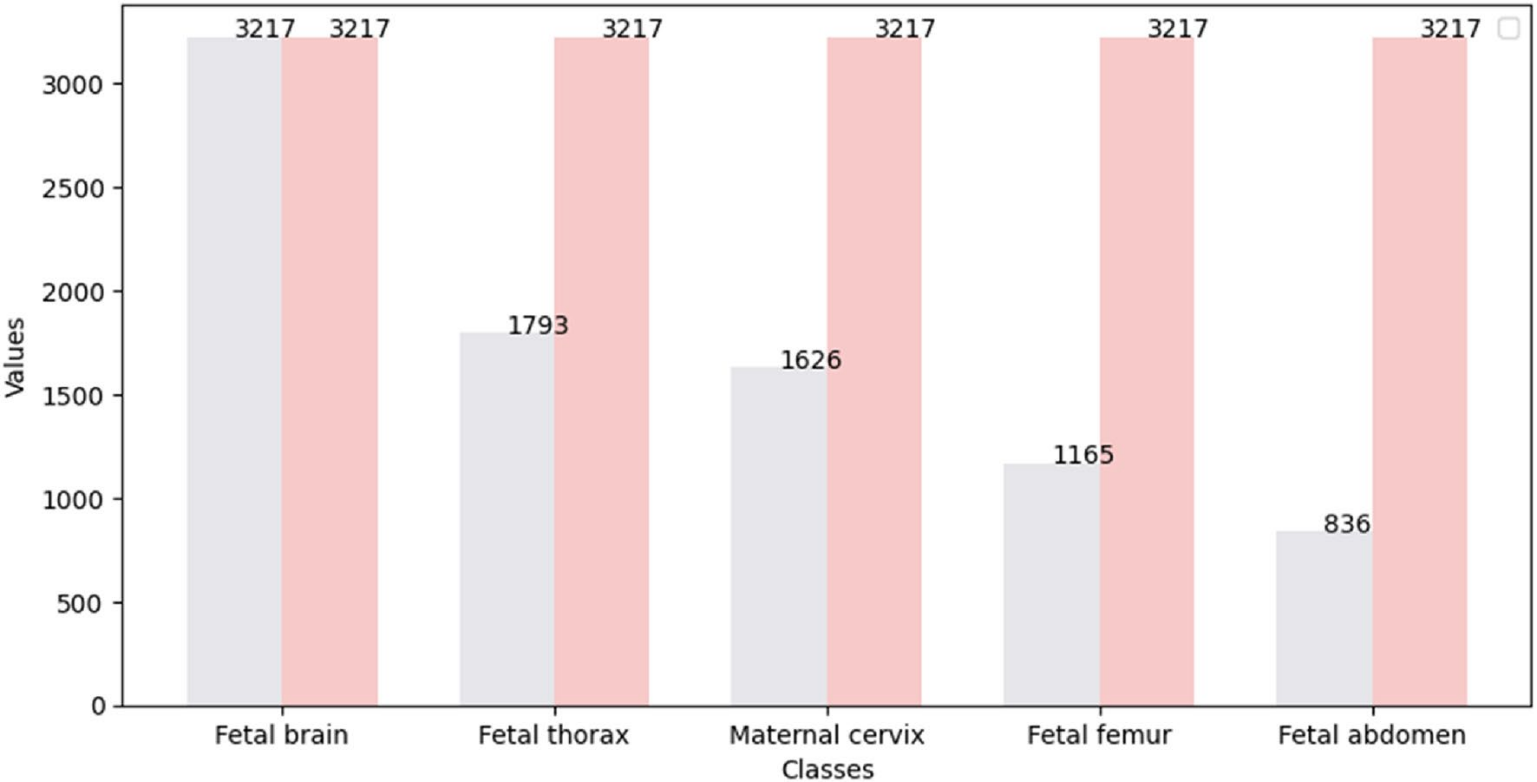
Maternal–fetal data distribution before and after data sampling.

Since a data imbalance and data insufficiency is noticed in Fig. 3, we proposed an oversampling method which is a data augmentation technique used to address class imbalance in machine learning models. This approach involves increasing the number of instances in the minority class by duplicating existing samples until it reaches a similar quantity as the majority class. Given that the fetal brain class has 3217 images, we employed an over-sampling technique for the other four classes in the combined dataset to ensure that each class has an equal number of images. This approach helps to balance the distribution of classes in the dataset, which can improve the accuracy of the model's predictions. Figure 5 shows the distribution of the dataset before and after the implementation of the data sampling technique. This technique will undoubtedly enhance the performance of the proposed models by addressing the issue of class imbalance or over-representation of certain classes in the dataset.

#### Data splitting

After applying data augmentation to the dataset, it becomes imperative to divide it into three subsets to facilitate training, validation, and testing. A widely used method involves randomly allocating 64% of the dataset to the training subset, 16% to the validation subset, and 20% to the testing subset. In this case, with a total of 16,085 images obtained after data cleaning and augmentation, the resulting split consists of 10,294 images in the training set, 2574 images in the validation set, and 3217 images in the testing set. This random splitting is important to prevent bias and overfitting. During the training phase, the deep learning model is trained using the training set. The hyperparameters are fine-tuned using the validation set to avoid overfitting. Finally, the model’s performance is assessed using the test set, which consists of previously unseen data. This evaluation ensures that the model can generalize well to new data and is not overfitting to the training set. By following this approach, the model proposed can be trained and evaluated effectively, leading to reliable results and informed decisions based on its performance.

### Model

This work aims to establish an efficient classifier to detect fetal ultrasound plans. Numerous studies have been used in their work; transfer learning which is a pertained model that was trained on ImageNet (14 M). Transfer learning^29^ is an artificial intelligence technique that has grown in popularity in recent years. It has been applied in image classification, natural language processing, and other machine learning applications. Instead of starting from scratch, the method involves using pre-trained models to solve new problems. This method is helpful since it conserves resources and time while producing excellent performance, especially when there is insufficient data to train a new model from scratch.

**Figure 6:**
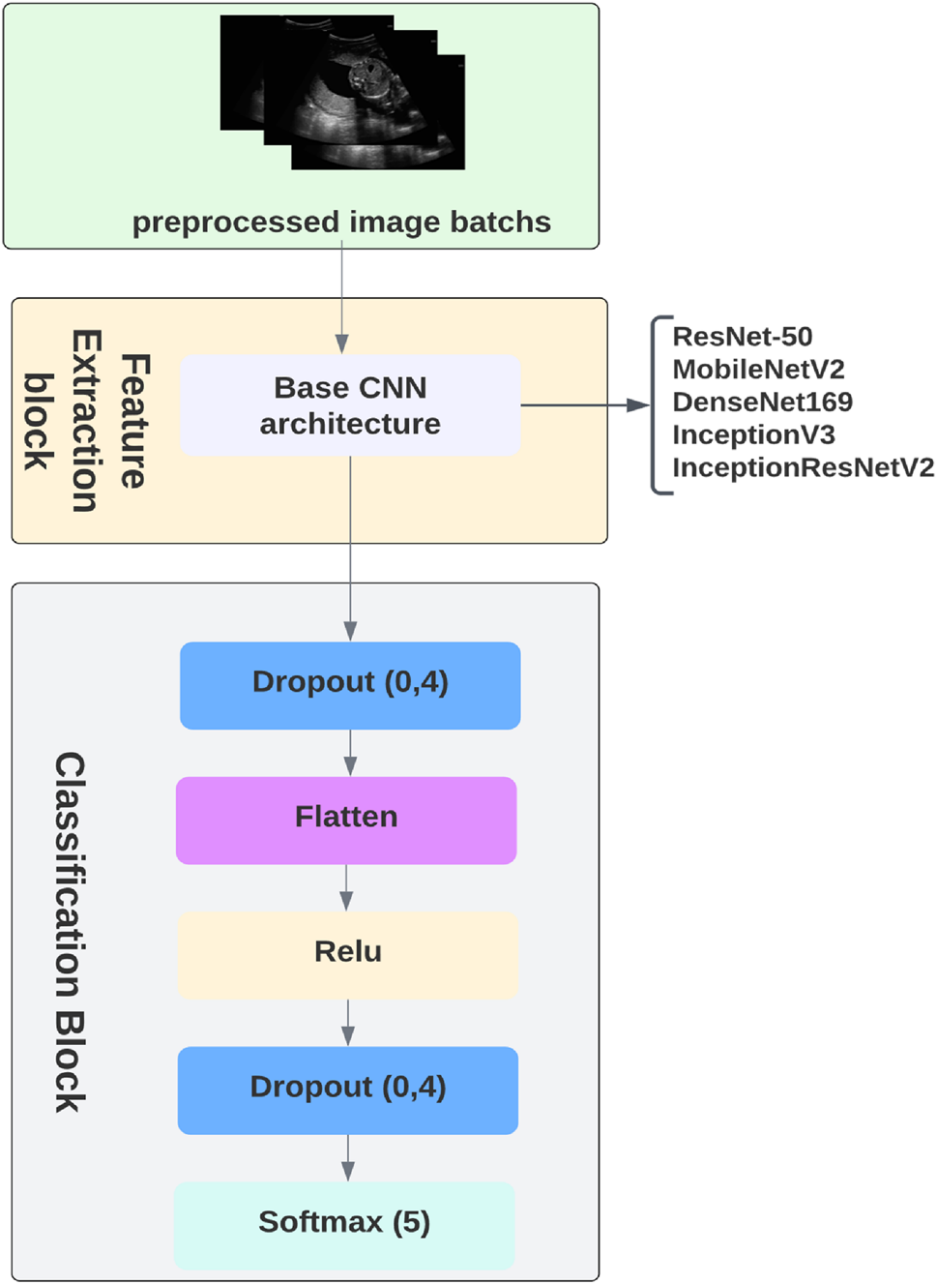
Presentation of models proposed for fetal organ classification.

In this paper, we proposed different algorithms based on a transfer learning strategy to classify maternal fetal ultrasound images. Those algorithms are based on the convolutional neural network (CNN) architecture. CNN architecture is characterized by the use of convolution layers, which allow for the extraction of features. To further enhance the benefits of the pre-trained weights, we conducted extensive trials to fine-tune the hyperparameters. After many trials, we found that incorporating a dropout technique with a rate of 0.4 effectively reduced overfitting and improved model generalization. Following this, we have applied Flatten and ReLu layers to the model, and the output from the ReLu layer is then directed to the output layer which utilizes the Softmax activation function to predict 5 classes. Figure 6 presented the models proposed in this study, in which we implemented the transfer learning models with the addition of new layers.

By leveraging the knowledge contained in pre-trained models, transfer learning can significantly reduce the amount of data and computation required to train accurate models, several CNN architectures were implemented in this work, namely:

- ResNet50^30^ is a deep convolutional neural network known for its residual connections, which enable the training of very deep networks. It contains 50 layers and has achieved state-of-the-art performance on many image classification tasks.
- MobileNet:^31^ is a lightweight convolutional neural network that is designed to be computationally efficient and is ideal for mobile and embedded applications. It uses depth-wise separable convolutions to reduce the number of parameters and computational costs while achieving good performance on many image classification tasks.
- DenseNet:^32^ is a convolutional neural network known for its densely connected layers, which enable feature reuse and reduce the number of parameters. It has achieved state-of-the-art performance on many image classification tasks while being computationally efficient.
- InceptionNet^33^ also known as GoogleNet, is a convolutional neural network known for its Inception modules and auxiliary classifiers, which enable the learning of multiple representations and improve accuracy. It has achieved state-of-the-art performance on many image classification tasks and is widely used in various applications.
- InceptionResNetV2^34^: is a very deep convolutional neural network that combines the Inception and ResNet architectures. It contains over 150 layers and uses Inception modules and residual connections to learn multiple representations of the same input and very deep representations.

After numerous trials and to further improve the accuracy of the neural network, the proposed models were trained using Adam optimizer with a learning rate of 0.001. Categorical cross entropy is proposed as a loss function, Table 2 summarizes the hyperparameters used in this approach. The model's optimal hyperparameter values are as follows: the ultrasound image input size is 155 × 224, with a batch size of 16, and a total of 60 epochs.

**Table 2:** Hyperparameters used in the proposed approach.

Hyperparameters	Optimized value
Optimizer	Adam
Learning rate	0.001
Loss function	categorical cross entropy
Train Validate and Test ratio	64:16:20
Batch size	16
Image input size	155 × 224
Epochs	60
Step size	1 m and 12 s

### Evaluation metrics

Several metrics are commonly used to assess the accuracy and robustness of classification models. The accuracy of a model is defined as the proportion of correct predictions made by the model out of the total number of predictions. This can be mathematically expressed in Eq. (1):

1 $${\text{Accuracy }} = \, \left( {{\text{TP}} + {\text{TN}}} \right)/\left( {{\text{TP}} + {\text{FP}} + {\text{FN}} + {\text{TN}}} \right)$$

where TP, TN, FP, and FN represent the number of true positive, true negative, false positive, and false negative predictions, respectively.

Another commonly used metric is precision, which is defined as the ratio of true positive predictions to the total number of predicted positive observations. This can be expressed in Eq. (2):

2 $${\text{Precision }} = {\text{ TP}}/\left( {{\text{TP}} + {\text{FP}}} \right)$$

The recall metric, on the other hand, is defined as the ratio of true positive predictions to all observations in the actual class, which can be expressed in Eq. (3):

3 $${\text{Recall }} = {\text{ TP}}/\left( {{\text{TP}} + {\text{FN}}} \right)$$

The F1 score, which is the harmonic mean of precision and recall, is a widely used metric for evaluating the performance of artificial intelligence models. It can be calculated in Eq. (4):

4 $${\text{F1 score }} = { 2}*\left( {{\text{Recall }}*{\text{ Precision}}} \right) \, / \, \left( {{\text{Recall }} + {\text{ Precision}}} \right)$$

Finally, the confusion matrix is a tabular representation of the various metrics mentioned above and is used to provide an intuitive understanding of the performance of a predictive model. A confusion matrix is shown in Table 3.

**Table 3:** Confusion matrix for a two-class problem.

		Actual	
		Positive: 0	Negative: 1
Prediction	Positive: 0	True Positive	False Positive
	Negative: 1	False Negative	True Negative

## Results and discussion

In this section, a descriptive analysis and a discussion of the proposed model’s results are presented. Furthermore, a comparative table (Table 6) is included to contrast the proposed work with other studies referenced in the related works. The presented experiment's results have obtained the usage of private cloud virtual machines that have the following characteristics: GPU Nvidia A100 with 24 GB Dedicated RAM, and 58 RAM associated with the instances for processing and cleaning the training data. The model results are discussed and evaluated in this section.

The objective of this study is to classify ultrasound images obtained during pregnancy using datasets collected from various countries and different types of scanning machines. This task was insured using various DL-based models which were assessed using different evaluation metrics such as accuracy, F1-score, AUC, and loss. In general, all of the proposed techniques produced high-quality results with different convolutional neural network (CNN) architectures, based on all the metrics. Specifically, the accuracy ranged from 99.26 to 99.78%, indicating that the model could correctly classify over 99.26% of the fetal ultrasound images. Additionally, the f1-score achieved outstanding outcomes, ranging from 99.62 to 99.77%, demonstrating that the model had an excellent balance between precision and recall. The AUC ranged from 99.26 to 99.78%, indicating that the model possessed high discriminatory power. The DenseNet169 model architecture performed best in terms of accuracy, f1-score, and AUC. Regarding the loss metric, all models generated excellent results. The InceptionV3 CNN-based model exhibited the lowest loss value, with 0.0233, which was roughly half the achieved loss of the DenseNet169. The other models also demonstrated respectable results, primarily InceptionResNetV2 and ResNet-50. Table 4 presents the results achieved for the transfer learning proposed models which are ResNet-50, MobileNetV2, DenseNet169, InceptionV3, and InceptionResNetV2 during evaluation.

**Table 4:** The achieved results during the evaluation phase: the evaluation includes all the proposed models.

CNN model proposed	Accuracy	F1-score	AUC	Loss
ResNet-50	99.26	99.62	99.26	0.1002
MobilenetV2	99.75	99.77	99.75	0.0356
Densenet169	**99.78**	**99.77**	**99.78**	0.0438
InceptionV3	99.67	99.68	99.67	**0.0233**
InceptionResNetV2	99.74	99.76	99.74	0.0420

Best performed results are in bold.

**Figure 7:**
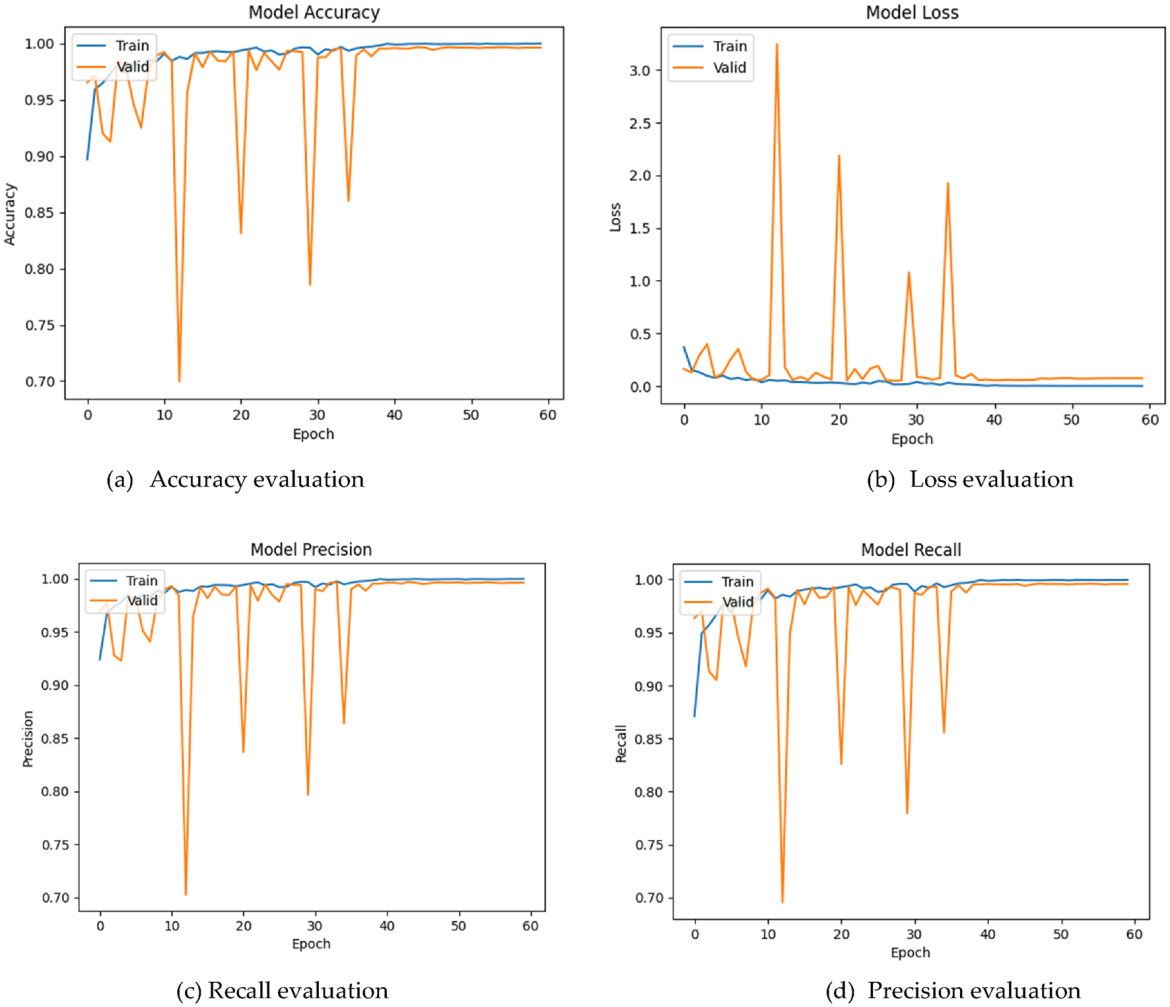
The evaluation of performance metrics throughout the training and validation phases, including accuracy (**a**), loss (**b**), precision (**c**), and recall (**d**).

Based on the evaluation of multiple classification models, the DenseNet169 architecture has been identified as the highest-performing model based on the F1-score metric. The F1-score is considered a comprehensive classification metric as it takes into account both precision and recall, which are important metrics for evaluating a model's ability to correctly identify positive and negative samples. This enables the proposed architecture to learn more complex and abstract features from the input images, conducting higher inference results. Overall, the DenseNet169 model's high performance on the F1-score metric suggests that it is an effective architecture for image classification tasks. Consequently, this model was selected for further investigation. During both the training and validation processes, the model achieved accuracy values of 99.97% and 99.61%, respectively, as shown in Fig. 7a. In terms of loss, the model produced values of 0.0007 and 0.0734 during the respective phases. as illustrated in Fig. 7b. Precision values were also high, with 99.98% and 99.61% obtained, respectively, as illustrated in Fig. 7c. Similarly, the model demonstrated high recall values of 99.96% and 99.57%, respectively, as shown in Fig. 7d. As depicted in Fig. 7, the metrics continue to oscillate for 37 epochs before reaching a stable state. This is because the model is still in the process of learning and adapting to the training data. During the early stages of training, the model is highly sensitive to small changes in the input data, which can cause the metrics to oscillate. As the model receives more training examples and adjusts its weights, it becomes more robust and better able to generalize to new data. This leads to a more stable performance, as reflected in the stable metrics observed in the later epochs of training. It is important to monitor these metrics during training to ensure that the model is learning effectively and to identify any potential issues, such as overfitting or underfitting, that may need to be addressed.

The performance evaluation of the DenseNet architecture in classifying the 5 maternal–fetal classes has yielded impressive results, with precision and recall metrics exceeding 99.21% across all classes. The high precision and recall values have contributed to an overall F1-score that surpasses 99.50%. These results demonstrate the exceptional accuracy and effectiveness of the proposed DenseNet169-based approach in tackling the classification task. Table 5 shows the classification report of the proposed method for the 5 classes.

**Table 5:** DenseNet169 classification report during the evaluation process.

	Precision	Recall	F1-score	Test samples
Fetal abdomen	0.9934	0.9967	0.9950	649
Fetal brain	1.0000	0.9921	0.9960	649
Fetal femur	0.9934	1.0000	0.9967	621
Fetal thorax	0.9984	0.9968	0.9984	644
Maternal cervix	0.9984	0.9984	0.9968	654

**Figure 8:**
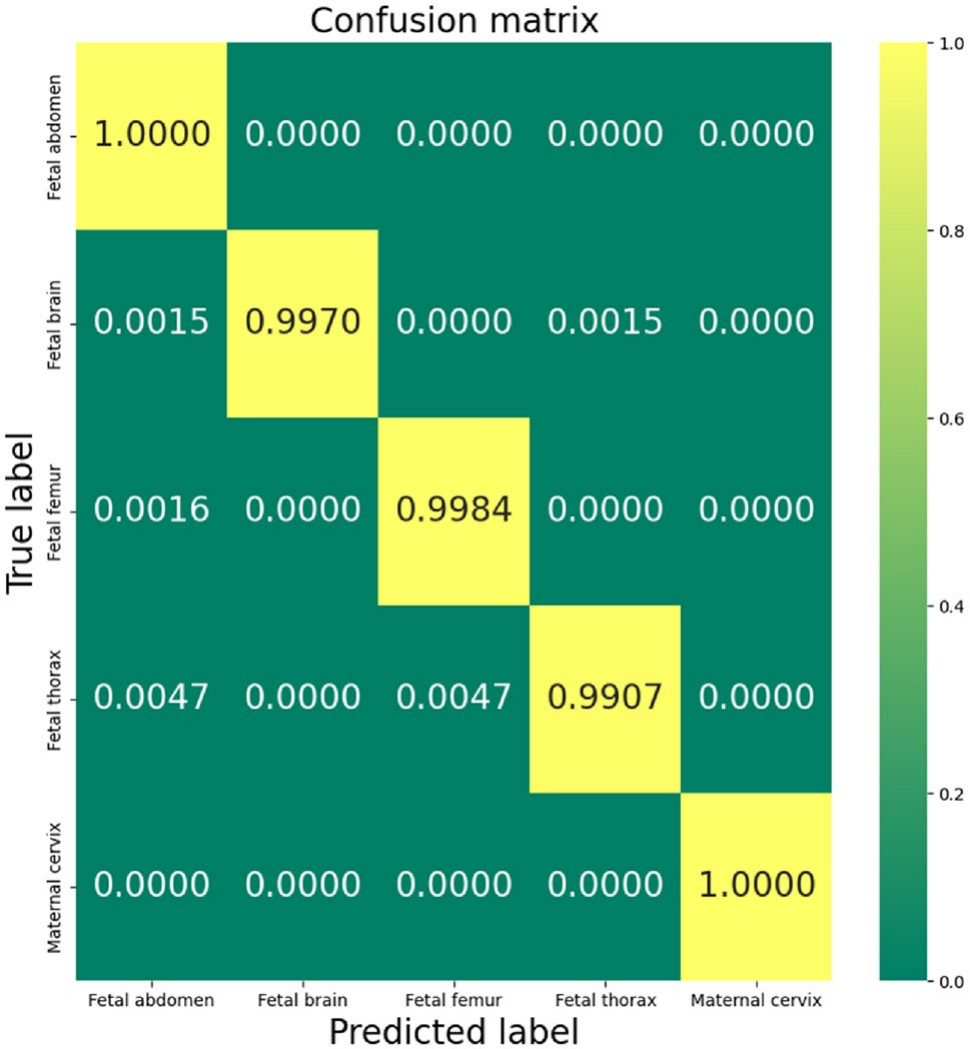
Confusion matrix for materno-fetal plans classification.

For the sake of further examination, the confusion matrix for the DenseNet169-based architecture was provided in Fig. 8, the fetal brain class is the only class with misclassification meaning the model that shows performant and stable classification results.

**Figure 9:**
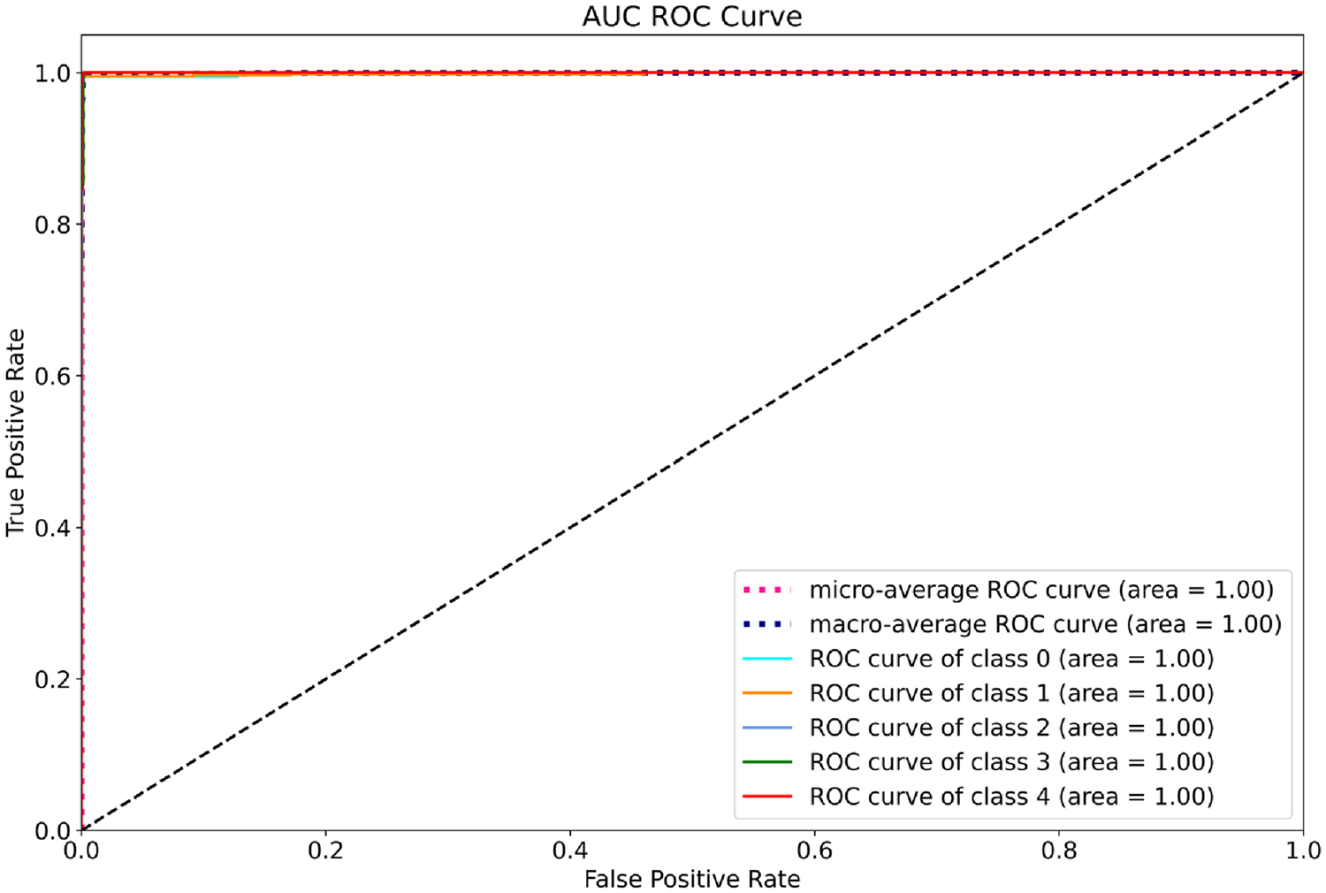
AUC-ROC curve of the model based on DenseNet169.

The AUC-ROC curves for the five-class classification provide a visual representation of the discrimination capability of the model for each class separately. Each curve shows the trade-off between sensitivity and specificity for a given class, with the area under the curve (AUC) indicating the overall performance of the model for that class. Overall, the mean AUC score across all classes provides a measure of the overall performance of the model, with higher values indicating better discrimination power. The description of the AUC-ROC curves for the five-class classification provides a comprehensive assessment of the model's performance, including details of the AUC scores, sensitivity and specificity for each class, and any patterns or trends observed in the curves. Figure 9 illustrates the AUC-ROC curves for the five-class classification of the model based on DenseNet169.

**Figure 10:**
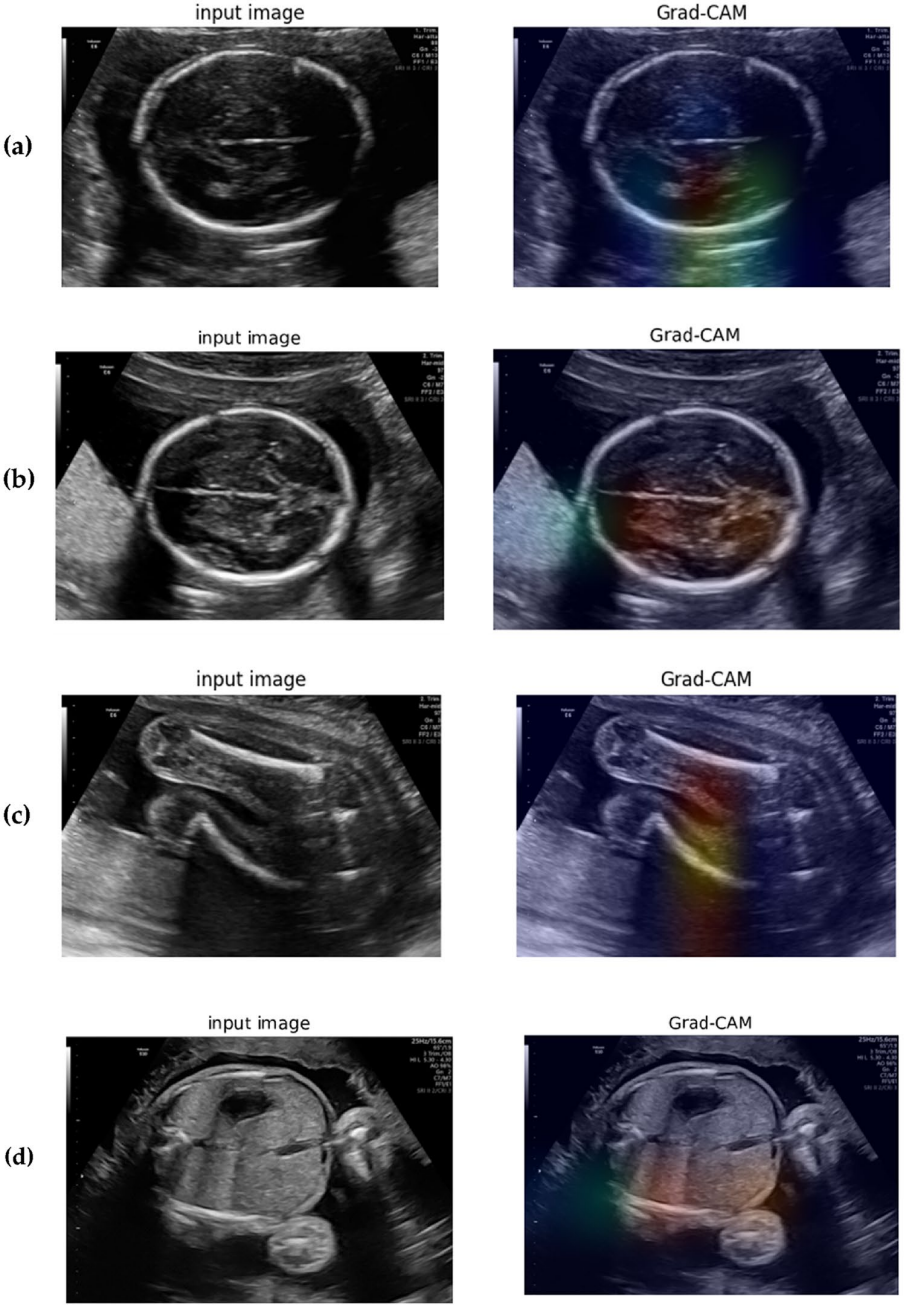
Visualization of fetal organ detection using Grad-CAM with DensNet model architecture. (**a**) and (**b**) Represent the class activation map for fetal brain detection, (**c**) shows the class activation map for fetal femur detection, and (**d**) shows the class activation map for fetal abdomen detection.

Grad-CAM^35^ is a powerful tool for visualizing and interpreting the results of deep learning models used for classification tasks. In the context of ultrasound materno-fetal images, where the goal is to classify images into one of five classes, Grad-CAM can provide insights into which parts of the image the model is focusing on in order to make its decision. Generating a class activation map by computing the gradient of the final convolutional layer allows us to determine the crucial image regions for the model's classification decision^36^. This approach aids in identifying distinctive features associated with each class, resulting in a more comprehensible understanding of the model's prediction process. Figure 10 depicts the Grad-CAM visualization results of DenseNet169, highlighting the proposed model's proficiency in identifying the fetal brain (Fig. 10a and b), fetal femur (Fig. 10c), and fetal abdomen (Fig. 10d) with impressive accuracy. The selected model takes about 32 ms to correctly detect the fetal planes. More importantly, this time can help clinicians minimize their detection time to improve patient outcomes. By using this model, clinicians can quickly and accurately identify the fetal planes, which can be critical in situations where time is of the essence, such as emergencies or cases where the fetus is in distress.

The proposed models, which are based on the Convolutional Neural Network (CNN) architecture and Transfer Learning strategy, have achieved superior performance when compared to other fetal classification methods described in the literature. Specifically, our model has surpassed traditional approaches that rely on the manual interpretation of ultrasound images by experienced clinicians, as well as other deep learning-based methods that use different network architectures and training strategies. Overall, the results indicate that the use of deep learning models based on CNN architecture can provide high accuracy in image classification tasks. The performance of these models depends on the size and quality of the dataset, as well as the specific architecture and optimization techniques employed. We have selected the DenseNet169 architecture, which has been shown to outperform approaches mentioned in the literature for the classification task and is effective in recognizing materno-fetal plans. By implementing a preprocessing process in which we enhance the public database, we were able to detect the presence of standard plans, which provide valuable information regarding fetal health and assist in making informed medical decisions. Therefore, it is likely that the Random Oversampling technique had a positive impact on the performance of the proposed models in the study, by oversampling the minority class, the model is trained on a more balanced dataset, which can lead to better generalization and prediction performance for ultrasound fetal organ. Using DenseNet169 on a dataset of 16,085 images, we achieved an accuracy of 99.97%, precision of 99.98%, and recall of 99.96%. These results demonstrate the effectiveness of the chosen model and dataset in accurately classifying ultrasound images. It is worth noting that the proposed approach utilized a smaller dataset compared to some of the other studies, indicating that the model's performance may improve with larger datasets. Our analysis of fetal ultrasound images provides a comprehensive assessment of fetal and maternal health, which is of great significance in the field of medical science. In Table 6, we present a comparison of our research with the studies presented in the related works section of the literature.

**Table 6:** Comparison of accuracy, precision, and recall with the state-of-art.

Author	Number of images	Model	Accuracy (%)	Precision (%)	Recall (%)
Qu et al.^19^	15,314	CNN	93	93	92
Kong et al.^20^	22,715	DenseNet	98.26	98.14	98.15
Liang et al.^21^	17,840	SPRNet	99	–	96
Our work	16,085	DenseNet169	99.97	99.98	99.96

## Conclusion

In conclusion, we propose deep learning models based on several popular CNNs, including InceptionResNetV2, InceptionNet, DenseNet, MobileNet, and ResNet50, for classifying materno-fetal classes. We aimed to improve a public dataset's quality by applying image cropping, data augmentation, and cleaning techniques. The proposed deep learning-based method provides an alternative tool for countries with insufficient healthcare systems especially since it was collected from different regions and different machines.

Our model achieved superior performance compared to previous literature works, demonstrating its effectiveness using various performance metrics. Specifically, our model achieved an accuracy of 99.78%, an F1-score of 99.77%, and an AUC of 99.78%. The results of our study demonstrate the effectiveness of transfer learning models in improving the classification of fetal ultrasound images.

In future work, we plan to augment the detected classes and apply filtering techniques to reduce speckle noise in ultrasound images. We also aim to explore the potential of incorporating additional features such as demographic information to improve the accuracy of the classification model. Furthermore, we plan to validate the performance of our proposed method in clinical settings to ensure its safety and reliability.

## Data Availability

The datasets used during the current study are available from the corresponding author on reasonable request.
